# Exploring the Role of Cellular Interactions in the Colorectal Cancer Microenvironment

**DOI:** 10.1155/jimr/4109934

**Published:** 2025-04-11

**Authors:** Jiadai Tang, Liuhan Chen, Xin Shen, Tingrong Xia, Zhengting Li, Xiaoying Chai, Yao Huang, Shaoqiong Yang, Xinjun Peng, Junbo Lai, Rui Li, Lin Xie

**Affiliations:** ^1^Department of Gastrointestinal Oncology, The Third Affiliated Hospital of Kunming Medical University, Yunnan Cancer Hospital, Peking University Cancer Hospital Yunnan, Kunming, Yunnan, China; ^2^Department of Head and Neck Surgery Section II, The Third Affiliated Hospital of Kunming Medical University, Yunnan Cancer Hospital, Peking University Cancer Hospital Yunnan, Kunming, Yunnan, China

**Keywords:** cellular and noncellular constituents, cellular and noncellular functions, colorectal cancer, tumor immune microenvironment

## Abstract

Colorectal cancer (CRC) stands as one of the tumors with globally high incidence and mortality rates. In recent years, researchers have extensively explored the role of the tumor immune microenvironment (TME) in CRC, highlighting the crucial influence of immune cell populations in driving tumor progression and shaping therapeutic outcomes. The TME encompasses an array of cellular and noncellular constituents, spanning tumor cells, immune cells, myeloid cells, and tumor-associated fibroblasts, among others. However, the cellular composition within the TME is highly dynamic, evolving throughout different stages of tumor progression. These shifts in cell subpopulation proportions lead to a gradual transition in the immune response, shifting from an early antitumor growth to a late-stage environment that supports tumor survival. Therefore, it is crucial to further investigate and understand the complex interactions among the various cell populations within the TME. In this review, we explore the key cellular components of varying origins, subpopulations with shared origins, and noncellular elements within the CRC TME, examining their interconnections and critical considerations for developing personalized and precise immunotherapy strategies.

## 1. Introduction

Although the ranking of tumor incidence and mortality has been fluctuating in recent years, colorectal cancer (CRC) consistently remains among the top five worldwide, with a significant disease burden in China irrespective of urban–rural stratification or gender differences [1]. Prevention, early diagnosis, and mired treatment of CRC are crucial for prognosis. However, the highly asymptomatic nature of CRC contributes to early metastasis, while the absence of personalized treatments postmetastasis is a major factor in poor prognosis [2, 3]. Over years of research, the role of the tumor immune microenvironment (TME) in tumor development and metastasis has become increasingly evident [4–6]. Although the TME mechanism remains incompletely understood, studies indicated that tumor cells could shape the microenvironment by recruiting or secreting various inhibitory cytokines to induce immunosuppression, evading immune surveillance through “immune escape,” often involving tumor-associated macrophages (TAMs) [7]. The diverse molecular subtypes of CRC lead to heterogeneous treatment outcomes, highlighting the need to reduce treatment-related toxicity by harnessing the autoimmune system against tumor cells [8]. This study aims to summarize current research on the cellular and noncellular components of the TME in CRC, offering a foundation for deeper investigation into the TME's intricate role in CRC progression.

## 2. Cellular Components and Their Roles in the CRC TME

**Figure 1:**
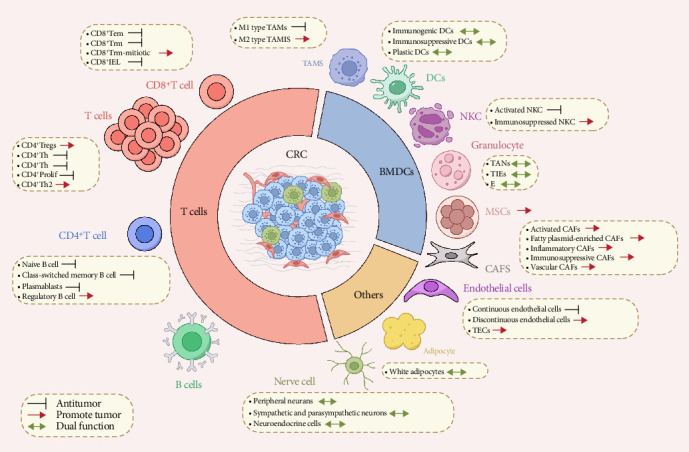
Constituents and functions of the TME cell components in CRC. CRC, colorectal cancer; TME, tumor immune microenvironment.

TME encompasses both cellular and noncellular elements [9, 10]. The CRC TME comprises tumor cells, innate and adaptive immune cells, mesenchymal cells, including cancer-associated fibroblasts (CAFs), and endothelial cells. Noncellular components include the extracellular matrix (ECM) [11] (Figure 1 and Table 1).

### 2.1. Lymphocyte Cell Population

#### 2.1.1. T-Lymphocyte

T cells play a central role in the immune microenvironment of CRC and are key factors in regulating tumor immune responses. The level of tumor-infiltrating lymphocytes (TILs) is closely related to tumor antigen load and patient prognosis. CD8^+^ T cells are cytotoxic and can recognize and kill tumor cells [12]. Studies have shown that the level of CD8^+^ T-cell infiltration contributes to tumor antigenicity, facilitating the transformation of “cold tumors” into “hot tumors,” thereby enhancing the benefits of immunotherapy [12, 13]. Conversely, cluster of differentiation 4 (CD4)^+^Foxp3^+^ regulatory T cells (Tregs) act as immunosuppressors in antitumor immunity, typically accumulating in the TME of CRC patients and entering peripheral blood as the tumor progresses, inducing immune tolerance and promoting immune evasion [14]. The ratio and functional status of T-cell subsets influence tumor development and treatment responses.

Cytotoxic T lymphocytes (CTLs) contain granules that are released upon specific interaction with target cells. These granules primarily consist of perforin, granzymes, and proteoglycans [15]. Killing of target cells occurs at a specialized contact site called the immunological synapse (IS), where regulated exocytosis of cytotoxic granules (CGs) takes place. CGs contain perforin-1 (Prf1), granzyme B (GzmB), and Fas ligand (FasL), which induce target cell death via apoptosis and necrosis. CGs are lysosome-related organelles that sharing similarities with endosomes and lysosomes [16]. These cytotoxic vesicles are transported along the cytoskeleton to the IS and fuse with the plasma membrane to release their contents. Due to the hazardous nature of CGs, their fusion is tightly regulated both spatially and temporally [17].

Research indicates that positive expression of programed death-ligand 1 (PD-L1) in tumor cells is closely associated with CD8^+^ T-cell infiltration, meaning that extensive cytotoxic T-cell infiltration is a driving factor for enhancing the antigenicity of CRC cells [14, 18, 19]. Activated effector T cells can not only secrete cytokines such as interferon-gamma (IFN-*γ*) and tumor necrosis factor-alpha (TNF-*α*) to enhance immune responses and recruit other cytotoxic cells like dendritic cells (DCs) and natural killer cells (NKCs) to collaborate in antitumor activities, but they can also differentiate into memory T cells under strong tumor antigen stimulation, allowing for rapid response and control of tumor recurrence and metastasis upon re-exposure to tumor antigens [20–24]. However, as the tumor progresses, CD8^+^ T cells may experience exhaustion, showing functional decline and decreased cytokine secretion, in which Tregs play a significant role [25, 26]. The proportion of Tregs is higher in CRC patients, and their infiltration is negatively correlated with tumor malignancy and prognosis [27]. Additionally, the increased surface expression of inhibitory receptors such as cytotoxic T-lymphocyte-associated protein 4 (CTLA-4), programed cell death-1 (PD-1), lymphocyte activation gene-3 (LAG-3), T-cell immunoglobulin and mucin domain 3 (TIM-3), and T-cell immunoglobulin and immunoreceptor tyrosine-based inhibitory motif domain (TIGIT) indicates T-cell exhaustion—a state of functional impairment characterized by gradual loss of effector function and changes in transcription and metabolism [28, 29]. PD-1 and CTLA-4 inhibitory molecules play a significant role in T-cell immune suppression and have shown strong prognostic predictive performance in CRC, representing a breakthrough point for immunotherapy.

The expression of LAG-3 on TILs in certain CRC tissues has been linked to advanced tumor stages, microsatellite instability-high (MSI-H), and poor prognosis, suggesting that LAG-3 could serve as a potential prognostic marker for CRC [30].  A recent study found that CD8^+^ T cells deficient in both PD-1 and LAG-3 exhibited enhanced tumor clearance and improved long-term survival compared to those lacking either PD-1 or LAG-3 alone. LAG-3 and PD-1 work together to promote T-cell exhaustion and are key regulators of TOX expression [31]. Additionally, the negative immune regulator TIM3 plays a role in tumor evasion. Elevated TIM3 expression reduces Th1 cell activity, diminishes the secretion of IFN-*γ*, and weakens other antitumor responses, thereby impairing the immune system's ability to fight the tumor [32].

Indoleamine 2,3-dioxygenase (IDO) is an enzyme that degrades tryptophan, an essential amino acid and is overexpressed in tissues such as the colon, intestines, and lungs. In CRC, tumor-driven regulation of IDO is linked to metastasis and is negatively correlated with T-cell infiltration [33]. IDO1 expression can be induced by inflammatory signals like IFN-*γ*, lipopolysaccharide, and TNF. Under pathophysiological conditions, IDO1 is highly upregulated in response to these inflammatory stimuli, and its overexpression is associated with improved detection across various cancer types, including CRC [34]. TIGIT is primarily expressed on activated T cells, Tregs, memory T cells, and NK cells. It exerts its immunosuppressive effect by binding to CD155 on DCs, thereby modulating cytokine production by DCs and indirectly influencing T-cell function. TIGIT also promotes T-cell exhaustion by competing with the costimulatory receptor CD226 for binding to CD155. In microsatellite unstable CRC (MSI-CRC), immune checkpoint molecules such as IDO1, LAG-3, and TIGIT are expressed at higher levels compared to microsatellite stable (MSS) CRC. Combining checkpoint inhibitors has shown to be more effective in MSI-CRC, enhancing immune cell cytotoxicity and targeting the immune evasion mechanisms associated with microsatellite instability [35].

Recently, Liu et al. [36] highlighted the pivotal role of RUNX proteins in regulating CD8^+^ T and CD103^+^CD8^+^ T cell–mediated antitumor responses within the TME of human CRC. Their study showed significant differential expression of RUNX genes between CRC and normal tissues. Patients with a higher proportion of infiltrating CD8^+^RUNX1^+^, CD103^+^CD8^+^RUNX1^+^, CD8^+^RUNX2^+^, CD103^+^CD8^+^RUNX2^+^, CD8^+^RUNX3^+^, or CD103^+^CD8^+^RUNX3^+^ T cells had better outcomes compared to those with lower proportions. The RUNX family—comprising RUNX1, RUNX2, and RUNX3—has long been recognized as essential regulators in developmental processes, and dysregulation of these genes has been linked to tumorigenesis and cancer progression.

The immune costimulatory molecule OX40 (CD134) is a promising novel target for CRC immunotherapy [37].

It is now ascertained that immune checkpoints include both inhibitors and promoters of T-cell activation. Inhibitory checkpoints, like CTLA-4, PD-1, and PD-L1, dampen T-cell activity while activating checkpoints such as LAG-3, OX40, and glucocorticoid-induced TNF receptor family-related proteins promote T-cell responses. Additionally, checkpoints-like IDO are involved in regulating T-cell metabolism. Blocking suppressive checkpoints, particularly PD-1, PD-L1, and CTLA-4, has demonstrated clear clinical benefits in patients with MSI-H or deficient mismatch repair (dMMR) metastatic CRC (mCRC).

Currently, significant progress has been made in the development of immune checkpoint inhibitors (ICIs) for solid tumors, and their combination with radiotherapy/chemotherapy has further improved clinical outcomes [38, 39]. However, their approval is limited to tumors with mismatch repair deficiencies or microsatellite instability, which is a major constraint [40, 41]. To address the deficiency of secondary TILs in immunotherapy that blocks T-cell inhibitory receptors, adoptive cell therapy (ACT) has emerged strongly [42–44]. As a highly personalized cancer treatment method, ACT involves genetically reprograming and expanding tumor-specific TILs, which are then reinfused into the patient to induce an autologous immune response to kill tumor cells. This approach has the advantages of eliminating certain T-cell suppressions and maintaining prolonged proliferation and cytotoxicity. However, the lack of universality of patient-specific TILs, along with the inefficiency of transporting TILs to the tumor center and the associated high toxicity, pose both economic and technical challenges for its clinical promotion in solid tumors. Therefore, a detailed classification and functional understanding of these T-cell populations can help guide the development of personalized immunotherapy strategies, improving treatment efficacy and patient survival rates.

#### 2.1.2. B-Lymphocyte

For a long time, T cells and NKCs have been considered the main mediators of antitumor immunity, while B-cell populations were thought to provide negligible antitumor effects merely as antibody producers [45]. Therefore, research targeting B cells for antitumor purposes has been quite limited. However, with the deepening understanding of the TME and advancements in single-cell sequencing technology, the role of B cells in the TME of CRC has gradually gained attention [46–48]. The infiltration composition of B-cell subpopulations in CRC tissues has been continuously clarified, and the infiltration levels of tumor-infiltrating B cells (TIL-Bs) vary at different stages of tumor development [49, 50]. In early primary CRC tissues, there is a rich presence of activated or terminally differentiated memory B cells and plasma cells compared to the peripheral blood of patients and nontumor patients. These cells work together to produce high-affinity antibodies that directly target tumor cell surface antigens or indirectly induce other immune cells to recognize and eliminate tumor cells, thereby enhancing tumor-specific immune responses and providing sustained and effective immune protection. In contrast, in late-stage tumors and metastatic sites, chronic inflammation caused by persistent tumor antigen immune responses leads to significant infiltration of regulatory B cells (Bregs), which promote immune tolerance and tumor escape by secreting regulatory factors such as interleukin 10 (IL-10) and transforming growth factor-beta (TGF-*β*), affecting tumor development and treatment [48, 51]. The mature B-cell subpopulations adjacent to tumor tissues are closely related to lymph node metastasis in stages I–III and low survival rates in CRC patients. TIL-Bs can also promote antitumor immunity in T cells through their antigen-presenting function, maintaining an immune “hot” TME [52, 53]. This indicates that these B-cell populations can combat immune editing and tumor heterogeneity by relaxing self-tolerance mechanisms, providing new therapeutic opportunities for cancer immunotherapy, and showing potential as biomarkers for prognosis prediction and immune targets in CRC progression.

Although the specific role of B cells in CRC tissues still requires further research, a large number of B cell–targeted drugs currently on the market or in development primarily focus on the treatment of autoimmune diseases and B-cell malignancies [54, 55]. Nonetheless, investigating their existence and functions offers fresh insights into the development of immune therapeutic strategies targeting B cells, with some progress already achieved. Researchers have utilized multidimensional omics data to map the genetic regulatory landscape of immune infiltration in CRC within the Chinese population, identifying a total of 568 immuno-QTLs [56]. These loci are significantly enriched in susceptibility regions for CRC and have been validated in the cancer genome atlas TCGA. Additionally, researchers have constructed a polygenic risk score (PRS) model, which effectively enhances the risk stratification efficiency for CRC and the identification of high-risk populations. In summary, B lymphocyte populations play an important role in the TME of CRC, and current research advancements provide new strategies and targets for immunotherapy in CRC. Therefore, gaining a more detailed understanding of tumor-associated B-cell subpopulations and their impact on tumor growth will facilitate the timely transition to antitumor clinical interventions.

### 2.2. Bone Marrow Cell Population

Bone marrow-derived cells (BMDCs), originating from the bone marrow, are a vital component in both tumor progression and immune response. TAMs, on the other hand, represent a predominant phenotype of myeloid cells infiltrating solid tumors, exerting significant influence on the TME and playing pivotal roles in tumor progression and metastasis.

#### 2.2.1. TAMs

TAMs are the predominant myeloid cells infiltrating solid tumors, playing a critical role in regulating the TME and driving tumor progression and metastasis. Their involvement in the clearance of apoptotic cells (cytophagy) aids in suppressing inflammation and contributes to immune evasion in CRC. Earlier studies have found poorer progression-free survival and chemotherapy response rates in the high TAMs group, which may be associated with inefficient drug distribution and chemoresistance due to TAMs [57]. Additionally, a high density of M2-type TAMs at the tumor center and infiltration margins is associated with poor differentiation and shorter disease-free survival. However, the same study suggested that a high density of pan-macrophages in these regions correlates with a more favorable prognosis [58, 59]. This apparent contradiction has sparked significant interest among researchers. With advances in technology and more refined studies, it has been observed that TAMs are typically induced by macrophage colony-stimulating factor (M-CSF), which differentiates monocytes into M0 macrophages. Subsequently, under the influence of specific cytokines, M1-type TAMs exhibit proinflammatory and antitumor effects. They can eliminate pathogens and stimulate antitumor immune responses by secreting proinflammatory cytokines such as TNF-*α*, IL-6, and IL-12. When appropriately activated, M1-type TAMs are capable of mediating phagocytosis and exerting cytotoxic effects to kill cancer cells [60]. However, in malignant tumors, TAMs are regulated by a variety of factors and mostly exhibit M2-type features, which promote tumor angiogenesis, alter CRC drug resistance, induce immune escape, and promote tumor proliferation and metastasis by inhibiting acute intense inflammation, secreting immunosuppressive factors, and regulating metastasis-related proteins [61, 62]. Furthermore, the recruitment of M2-type TAMs (marked by CD163) is significantly higher in metastatic CRC tissues compared to nonmetastatic ones. CD163 levels are also markedly elevated in mid-to-late-stage (stages III–IV) CRC tissues compared to early-to-mid-stage (stages I–II) [63–65]. In contrast, no significant difference was observed in the levels of M1-type TAMs (marked by CD68). These findings suggest that a high density of M1-type TAMs in the CRC stroma is associated with improved cancer-specific survival, whereas a high density of M2-type TAMs correlates with poorer survival outcomes in CRC patients [66, 67]. However, TAMs contribute far beyond their role in CRC progression. Acting as a bridge between inflammation and cancer, they induce early-onset CRC through metabolic reprograming, acidification of the TME, reduction of oxygen levels, and creating conditions favorable for tumor growth. Moreover, under the influence of various regulatory factors and chemokines, TAMs actively polarize toward the M2 phenotype. This polarization can even drive the secondary conversion of M1-type TAMs into M2-type TAMs, facilitating the aggregation of Tregs and increasing PD-1 expression, ultimately promoting immune evasion [60, 68].

As our understanding of the functions and mechanisms of TAMs deepens, distinct functional subtypes of M1 and M2 TAMs have been identified. These subtypes, when combined with other marker molecules, offer valuable potential for effectively predicting clinical outcomes. Targeted drugs based on different stages of CRC and subtypes of TAMs, such as probiotics that maintain microbial homeostasis, show strong potential for development, with the ultimate goal of inhibiting the polarization of M2-type TAMs. A study at Fudan University found that activation of the NOD1 receptor on the surface of TAMs could shift TAMs from the immunosuppressive M2 type to the CD8^+^ T-cell-activated M1 type and enhance the immune response, which provides a new strategy to enhance immunotherapy by regulating the polarization status of TAMs [69, 70]. On the other hand, metabolic remodeling can also be addressed. Studies have shown that M1-type TAMs predominantly rely on glycolytic metabolism for energy, while M2-type TAMs derive energy primarily through oxidative phosphorylation and fatty acid oxidation. This metabolic distinction suggests that reducing lipid uptake in TAMs by downregulating the scavenger receptor CD36 could decrease lipid accumulation within TAMs. Consequently, this reduction would impair fatty acid oxidation and oxidative phosphorylation, thereby inhibiting the polarization of TAMs toward the M2 state [71, 72]. The emergence of nanomaterials offers promising strategies for inhibiting tumor progression. For example, cationic polymers acting as TLR-4 ligands can activate the TLR-4 signaling pathway, stimulate the specific secretion of IL-12, and reverse the polarization of M2-type TAMs to the M1 phenotype [73]. These advancements provide novel perspectives for CRC immunotherapy, prognostic assessment, and the identification of potential therapeutic targets.

#### 2.2.2. DCs

DCs are a highly heterogeneous population of innate immune cells, and the recognition, capture, and processing of antigens by immature DCs in the peripheral blood and their presentation to naïve T lymphocytes are the most unique features of this subset [74–76]. A low level of antigenic stimulation can prompt DCs to transition from an immature to a mature state. This maturation reduces their antigen-capturing capacity while enhancing the expression of major histocompatibility (MHC) class I and II antigens, costimulatory molecules, and effector cytokines. These changes initiate T-cell immune responses and activate TILs and NKCs to exert cytotoxic effects. As a result, DCs play a pivotal role in inhibiting local tumor growth, proliferation, and metastasis [77, 78]. Compared with the normal population, the number and function of peripheral blood DCs subpopulations are impaired in CRC patients, limiting effector T-cell activity, and this effect correlates with patient prognosis. The infiltration of mature DCs is significantly higher in early-stage CRC primary tissues compared to metastatic tissues, with even higher levels observed in patients with microsatellite-unstable CRC. This suggests that DCs could represent a promising new target for cancer immunotherapy [79, 80]. However, tumor cells can suppress DC function by secreting immunosuppressive factors such as TGF-*β* and IL-10, which alter cytokine secretion patterns. This induces the production of immunosuppressive tumor-associated DCs (TADCs) and modifies metabolism to adjust the microenvironmental pH. These changes directly or indirectly impair DC functions, including antigen presentation, T-cell activation, and maturation, contributing to tumor cell evasion of immune surveillance and resistance to immune clearance. Therefore, understanding the inhibitory mechanisms of DCs by tumor cells is important for developing more effective immunotherapy strategies [81, 82].

DCs-based immunotherapy is in full swing: it has been found that vascular endothelial growth factor (VEGF) can impair the migratory ability and immune function of mature DCs through the VEGFR-2-mediated RhoA-cofilin1 signaling pathway, suggesting that blockade of this signaling pathway should be considered when administering DCs-based antitumor immunotherapy in the clinic in order to improve the clinical efficiency of treatment [83]. DCs vaccines have shown significant efficacy in clinical trials for a variety of cancers, especially in enhancing immune response and improving patient survival [84–86]. These vaccines activate the TILs response by activating the patient's immune system and prompting the presentation of potent tumor antigens and have been shown to have promising applications in a variety of types of cancers, including CRC, breast cancer, prostate cancer, and other types of cancers [87]. Additionally, researchers have developed a dual-response nanomedicine designed for programed antitumor immune activation. Delivered via the lymphatic system, this nanomedicine releases STING agonists in acidic lymph nodes, effectively activating DCs and T cells. It then accumulates in tumors to release aPD-1 for immune checkpoint blockade (ICB) therapy while promoting DC maturation and initiating robust antitumor immune responses [88]. These advancements not only offer valuable insights into the safety and efficacy of DC-targeted immunotherapy but also provide a crucial foundation for designing novel vaccines and enhancing existing therapeutic strategies. By conducting in-depth analyses of response variations among different patient groups and optimizing vaccine formulation and administration methods, the clinical efficacy of such treatments is expected to improve significantly in the future, offering new hope for advancing cancer therapy.

#### 2.2.3. NKCs

NKCs in bone marrow are one of the innate immune cell populations, accounting for less than other cells, about 5% or less, and varying with individual differences, age, health status, and immune status [89–91]. As cytotoxic members of the innate lymphoid cell (ILC) family, these cells play a crucial role in immunosurveillance and tumor clearance despite their low abundance. Circulating through blood, lymphatic, and nonlymphatic tissues, they are capable of mounting a rapid and robust response against cancer cells [92].

NKCs play a critical role in controlling cancer progression by killing mutant cells via death receptors and CGs. However, within the TME, NKCs often become exhausted, impairing their ability to surveil and respond to tumors, thus contributing to immune evasion. NKCs release CGs, such as perforin and GzmB, to induce tumor cell lysis and apoptosis [93]. Additionally, the Fc*γ* receptor III (CD16) on NKC recognizes antibodies bound to cancer cells, triggering NKC to release more CGs in a process known as antibody-dependent cell-mediated cytotoxicity (ADCC) [94]. Unlike CTLs, which require activation, NK cells constitutively express these granules [95, 96].

A study investigating the relationship between NKC activity and CRC risk found that NKC activity levels were lowest in the CRC group, intermediate in the colorectal adenoma group, and highest in the tumor-free group [82]. These findings suggest that NKC activity declines as the risk of CRC increases and that reduced NKC activity is associated with a higher incidence of CRC and colorectal adenomas. However, CRC tissues have high levels of chemokines such as CXCL10, CXCL8, CXCL5, and CXCL1, which are more likely to recruit T cells for aggregation than NKCs, which contributes to the general scarcity of NKCs in CRC tumor tissues and this phenomenon shows no correlation with the expression of the human leukocyte antigen MHC class I [97]. NKCs operate independently of MHC complex class I-mediated antigen presentation, highlighting their ability to function without relying on traditional MHC-based mechanisms [98]. This finding raises an intriguing question: could NKCs offer immunotherapeutic advantages over TILs that translate into clinical benefits? With ongoing research, it has been observed that activated NKCs in the TME exhibit potent cytotoxicity and killing activity. They can directly and independently destroy tumor cells by secreting cytotoxic and proinflammatory factors such as perforin, granzyme, and IFN-*γ*. Additionally, they enhance the antitumor immune response by recruiting other antitumor cell aggregates. Similar to T cells, a subset of NKCs can develop a memory-like effect, enabling them to quickly recognize and attack previously encountered tumor cells, further boosting the efficiency of immune clearance. Furthermore, epithelial-to-mesenchymal transition (EMT), a key driver of metastasis, can alter the immunogenicity of CRC cells, increasing their sensitivity to NKC-mediated cytotoxic cleavage and facilitating their elimination [99, 100]. However, the killing activity of NKCs is tightly regulated by various inhibitory signals, which can weaken their function or even turn them into “accomplices” of tumor cells. In the suppressive TME, functionally exhausted NKCs exhibit increased expression of PD-L1. This crosstalk between immune cells and tumor cells promotes EMT and suppresses the antitumor immune response [101]. As a result, the development of truly adaptive NKC-based therapies faces significant challenges. Nevertheless, achieving success in this area would represent a major milestone in the field of immunotherapy. Research on NKC-based tumor immunotherapies has advanced rapidly, encompassing a wide range of approaches such as chimeric antigen receptor (CAR) NKC therapy, cytokine mobilization, ICB, bi- or tri-specific killer cell engagers, and NKC-derived extracellular vesicles (NKEVs). For instance, in a human CRC NSG mouse model, NKG2D CAR-modified NKCs (NKG2D-DAP12CAR-NKCs) demonstrated enhanced IFN-*γ* secretion and a robust cytotoxic immune response, resulting in significant tumor suppression. Furthermore, local infusion therapy in patients with metastatic CRC has been shown to effectively prolong median survival times while demonstrating safety and feasibility [102]. In addition, the knockdown of TIPE2 using CRISPR-Cas9 technology can reverse the depletion state of NKCs, significantly enhancing their antitumor activity, suggesting that targeting TIPE2 represents a promising immunotherapy strategy [103]. Finally, NKEVs not only retain functional proteins from NKCs and exhibit antitumor activity both in vitro and in vivo but also effectively target tumor cells, inhibiting CRC growth and immune evasion through multiple mechanisms [104–106]. These advancements underscore the potential of NKCs in improving immunotherapeutic efficacy, driving the development of novel strategies, and leveraging NKC derivatives, paving the way for more effective cancer treatment options.

#### 2.2.4. BMDCs

Another significant population of innate immune cells with a high proportion in BMDCs is the granulocyte population. As key players in inflammation, granulocytes initiate, promote, and sustain inflammatory responses through chemotaxis, phagocytosis of pathogens, and the release of inflammatory factors, thereby protecting damaged tissues from infection. In addition, granulocytes facilitate the maturation and antigen presentation of DCs and activate NKCs and T cells, synergistically enhancing the immune response. Granulocytes are classified based on their specific cytoplasmic granule content into neutrophils (N), eosinophils (E), and basophils (B) [107].

Neutrophils release both cytoplasmic cytokines and factors stored in their granules. They contain three types of granules and secretory vesicles [108]. Azurophil granules hold MPO, which aids ROS production, and several serine proteases like neutrophil elastase, cathepsin G, and proteinase-3, which promote tumor invasion and proliferation [109]. Specific (secondary) granules contain lactoferrin and neutrophil collagenase (matrix metalloproteinase-8 [MMP8]), while gelatinase (tertiary) granules are rich in gelatinase B (matrix metalloproteinase-9 [MMP9]). Secretory vesicles release proteins integrated into the plasma membrane upon stimulation [108].

The neutrophil immune function relies on both the biogenesis of granules, which store antimicrobial proteins and their ability to generate an oxidative burst. Defects in either process can result in severe immunodeficiencies, such as neutropenia, neutrophil-specific granule deficiencies, or chronic granulomatous disease when oxidative burst is absent [110]. Granulocyte CSF (G-CSF) is the primary cytokine regulating granulocytopoiesis, and its absence impairs granulocyte production, leading to neutropenia and immune deficiency [111, 112]. Neutrophilic recruitment and activation can have broad effects on tumor cells and the microenvironment, including direct cellular injury from oxidant release, remodeling of the ECM, proangiogenic product release, and cross-signaling to other inflammatory cells and stromal constituents [113]. Neutrophils interact at these sites via cell contact or mediators such as reactive oxidants, granular proteins, and cytokines, which can prime or dampen antigen-dependent immune responses.

Eosinophils, traditionally known for combating parasites and contributing to allergic inflammation [114], produce a range of toxic granule proteins and proinflammatory mediators that can lead to tissue damage. They exert potent cytotoxic functions through the release of cationic proteins like major basic protein (MBP), eosinophil cationic protein (ECP), eosinophil peroxidase (EPX), and eosinophil-derived neurotoxin (EDN). Studies suggest that cytokines can influence eosinophil phenotype, determining their polarization and tumor immune responses. For example, Reichman and colleagues reported that in experimental CRC, intratumoral eosinophils exhibited an IFN-*γ*-related signature, which helped prevent the development of CRC in mice [115].

Although basophils make up a small proportion of circulating blood leukocytes, they are powerful immune effector cells. In CRC, low levels of circulating basophils have been associated with more advanced tumor stages (both T and N stages), venous and perineural invasion, and poorer patient survival [116]. One study suggested that basophils might be resistant to the immunosuppressive effects of Tregs (CD4^+^CD25^+^FoxP3^+^) and may even be activated by these cells instead [117]. Basophils are also capable of producing extracellular DNA traps containing mitochondrial DNA and granule proteins [118].

##### 2.2.4.1. Tumor-Associated Neutrophils (TANs)

Earlier studies highlighted the potential of granulocyte populations in CRC antitumor responses, but due to technical limitations, neutrophils (N cells) in vitro were unable to inhibit CRC cell proliferation. Instead, they reduced tumor cell adhesion and promoted their migration, leading to the hypothesis that the antitumor effects of TANs might be influenced by other components of the TME [119–121]. Initially, it was believed that TANs primarily functioned as tumor promoters in CRC, contributing to tumor invasion and angiogenesis through the production of MMP9, VEGF, and hepatocyte growth factor (HGF) [121]. However, as research advanced, it was discovered that TANs in CRC exhibit two polarized types within the TME. N1-type TANs possess antitumor properties, promoting T-cell activation, oxidative production, and tumor cytotoxicity. An increased proportion of CD16^+^TANs was associated with a better clinical prognosis. Additionally, high-density infiltration of myeloperoxidase-positive TANs was identified as an independent favorable prognostic factor for CRC [122, 123]. While the early inflammatory response is generally considered beneficial for antitumor immunity, prolonged chronic inflammation leads to changes in the release of inflammatory factors, oxygen levels, and acid-base balance in the TME, driving the conversion of TANs to the N2 type. In this process, TAMs and TADCs also play a significant role. Massively infiltrated N2-type TANs suppress the immune response by producing immunosuppressive and proinflammatory factors while also promoting tumor angiogenesis to support tumor proliferation and growth [124, 125]. In addition, CRC patients have been found to have a higher incidence of venous thrombosis, with markers of neutrophil extracellular traps (NETs), that is, Cit-H3, MPO, and cfDNA, significantly overexpressed in the peripheral blood of these patients [126–128]. This overexpression further promotes tumor cell migration to distant sites, facilitating tumor dissemination [129], which may explain the high infiltration of TANs [130] and their association with poor prognosis in CRC. Consequently, blocking the recruitment of TANs could serve as a potential breakthrough for CRC immunotherapy.

Although immunotherapeutic studies targeting TANs are still in the early stages, research is underway to enhance immunotherapy efficacy by reducing the number of N2-type TANs and inhibiting their accumulation in tumors [134, 135]. Existing studies have shown that combining oxaliplatin chemotherapy with immunotherapy can recruit N1-type TANs and enhance the effects of lipopolysaccharide A immunotherapy [136]. In contrast, the accumulation of N2-type TANs in tumors is linked to responses to anti-PD-1 therapies, but excessive accumulation may impair the effectiveness of ICIs. Single-cell sequencing analyses of CRC samples from patients undergoing PD-1 blockade therapy at multiple time points have revealed insights into the evolution of local and systemic immunity, as well as the coordinated cellular responses associated with therapy, helping to pinpoint optimal drug administration timing and developing new strategies to improve immunotherapy responses in CRC patients [137].

##### 2.2.4.2. Tumor-Infiltrating Eosinophils (TIEs) and Tumor-Infiltrating Basophils (TIBs)

In recent years, the focus has primarily been on the role of TANs, often overlooking the importance of other granulocyte types. TIEs in CRC are commonly clustered at the center of the tumor and at the front of invasive tumors. They can directly kill tumor cells through the release of cytotoxic compounds and eosinophilic extracellular traps or indirectly by activating other immune cells and cytokines to exert an antitumor effect. A high number of TIEs is independently associated with a better prognosis as predicted by TNM staging. Their cytotoxic effects are distinct from those of CD8^+^ T cells, as TIEs are more specifically targeted to solid tumors rather than lymphomas [138, 139]. TIBs are a rare subpopulation of leukocytes whose primary function involves the modulation of immune responses and allergic reactions. Although their role in CRC has been relatively less studied, under certain experimental and clinical conditions, TIBs, activated by IL-33 and their mediators, can play an antitumor role. They achieve this by secreting cellular particles such as histamine and inflammatory factors, which enhance inflammatory responses and recruit TILs into tumors to promote apoptosis [140, 141]. Additionally, some clinical studies have found that the number of circulating granulocytes in peripheral blood, regardless of the specific type, can reflect the level of tumor infiltration to some extent. This finding offers new hope for simplifying the detection process. However, further research is needed, as granulocyte subpopulations may have more therapeutic potential than NKCs due to their target specificity.

#### 2.2.5. Mesenchymal Stem Cells (MSCs)

The most specialized cell population among BMDCs is the multipotent stem cell, known as MSCs. These cells possess the unique abilities of self-renewal, multilineage differentiation, and tissue repopulation. MSCs can be isolated from various tissues and are extensively utilized in tissue repair, graft-versus-host disease treatment, autoimmune disease management, and oncology research [144, 145]. The classification and functional mechanisms of MSCs in CRC remain a topic of debate. Insights from single-cell transcriptome analyses have identified five distinct MSC subpopulations: (a) Stem cell-like subpopulations, which are involved in self-renewal and differentiation, providing essential support for sustained tumor growth. (b) MSC subpopulations, characterized by their ability to differentiate into various cell types, contribute to the TME and influence tumor invasiveness and metastatic potential. (c) Monopotent adipose precursor cells, associated with fat metabolism, potentially impacting CRC progression through energy metabolism regulation. (d) Bipotent chondrogenic-osteogenic precursors linked to bone metastasis and tumor-associated bone disorders. (e) Chondrogenic precursor cells, which possess immunomodulatory properties and may play a role in immune system regulation [146].

The identified MSC subpopulations highlight their dual role in CRC progression. On the one hand, MSCs can synergistically promote tumorigenesis by recruiting protumor inflammatory factors that induce the differentiation of CAFs [147, 148]. This contributes to tumor growth, invasion, migration, and metastasis, driven by their proangiogenic potential. Furthermore, MSCs subtly reshape the peritumoral cellular and stromal landscape, fostering the formation of an inhibitory TME, increasing drug resistance, tumor recurrence, and suppressing antitumor immunity, ultimately worsening patient prognosis [149, 150]. On the other hand, MSCs exhibit antitumor effects, particularly in the early stages of tumor development. During the initial phases of CRC, MSCs can migrate to the colorectal region, mitigate chronic inflammation, and regulate imbalances in the intestinal flora. By recruiting and activating Tregs, they can help inhibit tumor progression and contribute to antitumor responses [131, 132, 151]. MSCs have demonstrated remarkable tumor-homing properties, enabling precise migration to tumor sites and the targeted release of therapeutic agents. Their derived exosomes are effective drug carriers, positioning MSCs as potential nanomaterial delivery systems. This capability significantly enhances the efficacy of chemotherapeutic drugs while reducing their adverse effects, improving both the safety and effectiveness of CRC treatments. MSCs' unique tumor tropism has already led to their inclusion in clinical trials, with over 50 drugs currently registered for CRC treatment [133]. However, it is important to note that the role of MSCs can vary across different stages and subtypes of CRC. This duality underscores the need for continued research into their mechanisms and interactions within the TME. A deeper understanding of MSCs roles will be crucial for refining therapeutic strategies and improving the accuracy of patient prognosis.

### 2.3. CAF Populations

CAFs represent the most prevalent fibroblast subset within tumor tissues and are integral components of the TME. They contribute to tumor progression by remodeling the ECM, fortifying physical defenses, and secreting both inhibitory cytokines and proinflammatory factors. CAFs also modify the pH and metabolic landscape of the TME, hinder immune cell infiltration and activity, and shield tumor cells from immune surveillance. Furthermore, CAFs create barriers that obstruct drug and immune cell access, diminishing treatment efficacy and fostering chemoresistance. These activities collectively facilitate tumor cell proliferation, invasion, and metastasis [152–155].

CAFs establish the structural “framework” of the TME, while ECs ensure the integrity and functionality of blood vessels. In the TME of CRC, ECs play critical roles in regulating tumor angiogenesis, blood supply, and nutrient delivery, as well as contributing to T-cell activation, proliferation, and immune response modulation [156, 157]. Tumor-associated ECs also promote the formation of lymphatic structures at tumor sites and are linked to improved outcomes with ICIs. During angiogenesis, ECs rely on glycolysis for energy production, creating a hypoxic microenvironment that impairs immune cell function. Meanwhile, CRC cells sustain rapid proliferation through the Warburg effect. Together, CAFs and ECs collaborate to shape the TME, with each playing an essential role in its development and maintenance [60, 158–162].

Recent advances in cancer research have led to the development of mesoporous silica nanoparticle (MSN)–core-based nanocarriers designed for dual purposes: reprograming the metabolism of CAFs and eradicating cancer cells [163]. These nanoparticles enable the precise delivery of cytotoxic drugs or vaccines to selectively target CAFs, leveraging specific surface markers to eliminate them or revert activated CAFs to a normalized phenotype. Additionally, they address CAF-related metabolic pathways, downstream signaling, effector molecules, and ECM proteins. This approach mitigates the tumor-suppressive microenvironment, improves anticancer drug penetration, and fosters immune cell activation and infiltration [77].

Beyond the well-characterized cell types in the TME, adipocytes and neurons also play pivotal roles [164–166]. Adipocytes supply essential nutrients and bolster tumor cell metabolism and growth by secreting lipid metabolites, hormones, and cytokines. They also suppress antitumor immune cells that depend on aerobic glycolysis for energy. A dynamic exchange of metabolites and amino acids occurs between adipocytes and cancer cells, further supporting tumor progression. Moreover, adipocytes contribute to low-grade chronic inflammation favorable for tumorigenesis by releasing chemotactic factors that recruit BMDCs, leading to the generation of immunosuppressive cell populations [78, 167].

In response to the physical stress of the TME, adipocytes can dedifferentiate, gaining long-term self-renewal capabilities. These dedifferentiated cells can further differentiate into myofibroblasts, which in turn enhance tumor growth and migration [79]. Neurons also play a significant role in the TME by modulating the behavior of tumor and immune cells [168–170]. They achieve this through the release of neurotransmitters and neuropeptides, which influence cellular activity and infiltration, thereby impacting tumor progression and immune dynamics [171].

Additionally, metabolites produced by the gut microbiome, such as isovaleric acid, can activate the promoter of the tryptophan hydroxylase (Tph) 2 gene in serotonergic neurons within the gut, leading to upregulated Tph2 expression [172]. Tph2 drives serotonin production, which interacts with receptors (HTR1B/1D/1F) on CRC stem cells, fostering their self-renewal and tumorigenesis. This highlights a significant interaction between gut neurons and intestinal tumors, emphasizing the roles of gut neurons and microbiota in regulating CRC stem cells. These insights also suggest a potential therapeutic target for the prevention and treatment of CRC [173].

The above findings underscore the dynamic nature of the TME, where the components are continuously changing. The interaction network between different immune cell populations regulates the intensity and nature of the immune response, influencing the processes of tumor immune escape and growth.

### 2.4. Others

#### 2.4.1. Extra Cellular Matrix

The ECM serves as a crucial structural support for CRC cell growth and migration within the TME [174, 175]. Its composition and regulation, influenced by CAFs and their secreted factors, have garnered significant interest in CRC research [176–178]. The ECM is primarily composed of the following components: (a) Collagen: as the most abundant ECM protein, collagen provides tissue strength and reduces deformation. In CRC, it indirectly promotes tumor progression by activating the PI3K/AKT pathway through integrin *α*2*β*1 signaling. (b) Fibronectin (FN): a protumorigenic, noncollagenous protein found in the ECM and basement membrane, predominantly produced by liver and vascular endothelial cells. FN induces EMT and promotes CRC progression via the PI3K/AKT pathway. Its levels can also serve as a prognostic marker. (c) Laminin (LN): an essential component of the basement membrane, in conjunction with type IV collagen. LN derivatives enhance CRC liver metastasis, promote branching angiogenesis, and inhibit the Notch signaling pathway. (d) Hyaluronic acid (HA): this glycosaminoglycan binds to collagen, aiding in hydration and tissue compressive resistance. Interestingly, HA overexpression suppresses CRC liver metastases, underscoring its nuanced role. (e) Periostin (POSTN): a protein secreted by fibroblasts that plays a key role in regulating intercellular adhesion. POSTN is heavily involved in metastatic invasion and is a critical ECM component. (f) Follistatin-like 3 (FSTL3): overexpressed in CRC tissues, FSTL3 is associated with lymph node metastasis and resistance to chemotherapy. However, it exhibits sensitivity to immunotherapy, offering a potential therapeutic target.

These ECM components not only provide structural integrity but also actively influence tumor progression, metastasis, and therapeutic responses. Understanding their roles could unlock novel strategies for CRC treatment and prognosis. Abnormal deposition and cross-linking of the ECM result in increased matrix stiffness within the TME [179, 180]. This alteration not only provides structural support to the tumor but also creates a multifaceted barrier with significant implications: (a) Physical barrier formation: increased ECM stiffness physically compresses microvessels, leading to reduced blood flow and contributing to the development of a hypoxic environment. (b) Impact on therapeutic efficacy: hypoxia and physical barriers hinder neovascularization, impair the delivery of chemotherapeutic agents and reduce the penetration effectiveness of radiation therapy. (c) Immune modulation: the altered ECM stiffness influences the infiltration, activation, and function of immune cells within the TME. This regulation significantly impacts the success of immunotherapeutic approaches.

These factors highlight ECM stiffness as a critical determinant in CRC progression and treatment outcomes, underscoring its role as a potential therapeutic target to improve drug delivery, radiation efficiency, and immune cell function in the TME.

In CRC, the abundance and composition of the ECM are strongly associated with patient survival, with a negative correlation observed between ECM levels and prognosis. ECM proteins contribute to tumor progression and metastasis by promoting tumor cell proliferation and survival [181, 182]. However, studies have primarily established a link between elevated ECM proteins derived from tumor cells (parenchyma) and poor patient survival, while the relationship between ECM proteins from mesenchymal cells and survival remains uncertain and lacks a clear explanation.

It is well-established that increased ECM stiffness exacerbates the immunosuppressive environment within the TME, reducing the effectiveness of immunotherapy. One mechanism involves the activation of the ROCK-myosin II signaling pathway, which weakens CRC cell immunogenicity and induces the conversion of TAMs to the anti-inflammatory M2 phenotype, thereby impairing immune responses [183]. Additionally, studies have shown that DCs cultured on softer substrates demonstrate higher differentiation rates, whereas DCs grown on stiffer substrates show impaired differentiation and antigen presentation, affecting their ability to activate TILs. The remodeling of ECM also forms a physical barrier that hinders the migration of TILs, induces hypoxia, and weakens the function of NK cells [184].

Current therapeutic strategies targeting ECM hardness, such as matrix modulators, inhibitors of mechano-transduction pathways, and biomaterials designed to mimic the ECM, aim to enhance tumor immunotherapy. These strategies seek to improve drug and immune cell infiltration, modulate immune cell function, and reduce the immunosuppressive TME. However, inconsistent efficacy and a lack of conclusive evidence present significant challenges, requiring further exploration to optimize these approaches.

#### 2.4.2. Complementary System

The complement system is a crucial component of the body's immune defense, consisting of approximately 30 different plasma proteins typically found in an inactive state in the peripheral blood. The system can be activated through three distinct pathways: the classical pathway, which is antibody-dependent and involves the binding of pathogens to antibodies; the alternative pathway, which is antibody-independent and directly activates on the surface of pathogens; and the lectin pathway, which depends on specific glycans. Once activated, complement components perform vital functions, including marking pathogens for destruction, attracting immune cells, and directly killing infected cells. The complement system plays a central role in immune responses, such as anti-infective defense, inflammation, and tumor immune surveillance.

Both tumor cells and stromal cells in the TME of CRC produce abnormal complement proteins, leading to the aberrant activation of the complement system. Evidence suggests that these complement components may have an immunosuppressive role in the TME, acting as a bridge between tumor progression and immune responses that suppress tumor activity. For instance, C1q, an initiator protein in the classical complement pathway, has been linked to poor prognosis in CRC patients, with significant deposition in the TME. Studies have shown that C1q induces a tolerogenic or immunosuppressive phenotype in TAMs, favoring the polarization of M2-type TAMs and promoting their chemotaxis toward CRC tissues. This process correlates with the upregulation of PD-L1 and PD-L2, as well as reduced T-cell proliferation.

In contrast, other complement proteins, such as C3a and C5a, act as allergenic toxins that promote tumor metastasis and tumor-associated angiogenesis by activating CD4^+^ T cells. Specifically, C5a has been shown to facilitate CRC metastasis by stimulating the secretion of leukotriene B4 from epithelial and endothelial cells, as well as promoting the recruitment of TANs. Furthermore, C5aR1 can inhibit Th1 production and enhance the M2 polarization of TAMs [185, 186]. These findings emphasize the complex roles of the complement system in CRC, highlighting its involvement in tumor progression and immune modulation while also offering new insights and potential targets for CRC diagnosis and therapy.

Inhibiting the activation of the downstream complement system through complement inhibitors has been shown to modify the natural progression of diseases, improving conditions such as renal impairment, thrombosis, and pulmonary hypertension. This approach is considered an important strategy for the treatment of CRC [187]. Targeting specific complement-regulating proteins offers another potential therapeutic strategy. For example, a current Phase I clinical trial has demonstrated that combining C5aR1 antagonists with PD-1 inhibitors in the treatment of advanced solid tumors produces promising synergistic effects [188]. However, while this combination shows potential, there is currently insufficient evidence in CRC, making it a promising avenue for future research.

### 2.5. Microsatellite Instability and its Impact on the CRC Microenvironment

Molecular alterations in CRC can be broadly classified into three main categories: (a) Chromosomal instability (CIN), (b) MSI, and (c) CpG island methylator phenotype (CIMP), the latter of which involves the silencing of gene function through aberrant hypermethylation [142, 189, 190]. CIN-positive CRCs are characterized by structural and numerical changes in chromosomes, along with increased mutation rates in both tumor suppressor genes and oncogenes. Notably, mutation rates at single nucleotides are higher in MSI-positive CRCs than in CIN-positive CRCs. On the other hand, 15% of CRCs exhibit MSI due to defects in the DNA MMR system [191]. dMMR results from mutations in MMR genes, leading to a loss of function in one or more MMR proteins (such as MLH1, MSH2, MSH6, and PMS2), which causes errors during DNA replication. MSI occurs when there is an accumulation of nucleotide insertions or deletions in the genome and is classified into three categories: MSI-H, MSI-L, and MSS. CRC patients are generally divided into two groups: dMMR/MSI-H and pMMR/MSS or MSI-L (referred to as the pMMR/MSS group). Microsatellite status is associated with a range of clinicopathological features and plays a key role in drug resistance and patient prognosis. CRC patients with different microsatellite statuses show variations in the composition and distribution of immune cells and cytokines within their TMEs.

CRC patients, especially those with dMMR/MSI-H tumors, show significantly greater sensitivity to ICIs compared to those with MSS or MSI-L tumors, resulting in substantial clinical benefits from immunotherapy. The MSI status plays a key role in shaping the TME of CRC, influencing the effectiveness of ICIs. Recent findings suggest that MSI-H CRC tumors have a higher tumor mutational burden (TMB)—greater than 12 mutations per 10^6^ DNA bases—compared to MSS/MSI-L tumors, which typically have a lower TMB (fewer than 8 mutations per 10^6^ DNA bases) [192]. Additionally, MSI-H tumors are characterized by greater immune cell infiltration, particularly TILs and type I interferons, both of which are associated with a more favorable prognosis [193]. In contrast, a TME dominated by Th17-type cells and IL-17 is linked to poorer outcomes [194]. However, despite these insights, most studies have not thoroughly explored the differences in the immune microenvironment between MSI-H and MSS/MSI-L CRCs. The immune-inflamed MSI microenvironment is characterized by a higher infiltration of antitumor immune cells, including adaptive immune cells (such as T and B lymphocytes) and innate immune cells (like DCs, macrophages, and NK cells), compared to the immune-desert MSS tumors [195]. In CRC, mutations or activation of the MYC and RAS pathways can lead to the expression of the chemokine CCL9, which fosters an immunosuppressive environment, inhibiting the accumulation of cytotoxic NK cells and T cells around the tumor [196]. CRC patients with different MSI statuses show distinct immune cell and cytokine compositions within their TMEs.

**Figure 2:**
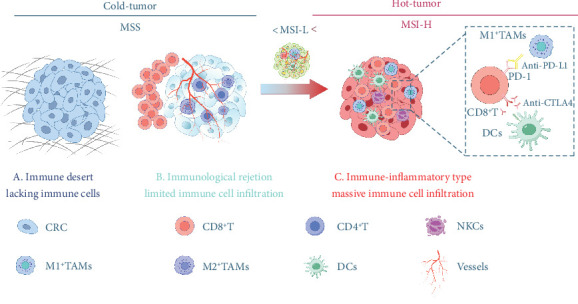
Microsatellite Instability and Its Impact on the CRC TME. CRC, colorectal cancer; TME, tumor immune microenvironment.

Studies on immune and stromal cell types using transcriptome-based cell-type quantification methods and pathway analyses have revealed distinct patterns based on MSI status in CRC. In the MSI group, IFN-*γ* and CD8 T effector gene signatures were highly activated compared to the MSS group. This activation correlates with increased CD8 T-cell infiltration, as well as upregulation of PD-L1, PD-L2, and p-STAT1, highlighting the unique immune landscape of MSI tumors. These findings suggest that the distinct TME of MSI and MSS tumors plays a critical role in immunotherapy outcomes, emphasizing the need to understand both TME dynamics and tumor characteristics. Consequently, a transcriptome-based MSI predictor that integrates TME and molecular pathway features in CRC is essential. Additionally, MSI-H tumors exhibit increased secretion of key cytokines such as TNF, perforin, granzyme, IL-1, IL-6, and IFN-*γ* within the TME. These cytokines regulate the immune “activation” or “inhibition” states of the TME [197]. The presence of various inflammatory mediators contributes to the formation of an inflammatory TME, where continuous inflammatory stimulation can lead to T-cell exhaustion. This exhaustion is associated with the upregulation of inhibitory receptors like PD-1, CTLA-4, TIM-3, and LAG-3, further complicating the immune response in these tumors [40]. These immunosuppressive receptors bind to their corresponding ligands within the TME, modulating the antitumor immune response. Research has shown that increased interferon expression is linked to a better prognosis, as it can stimulate the secretion of chemokines and promote adaptive immune responses. Consequently, high concentrations of TILs in MSI-H CRC patients are associated with improved survival outcomes. In contrast, the TME of MSS CRC patients typically exhibits an immune rejection or immune desert phenotype, characterized by low TIL infiltration and a lack of CTLs or insufficient CTL activity. As a result, strategies aimed at reshaping the TME and enhancing TIL infiltration could represent promising therapeutic avenues for MSS CRC patients (Figure 2 and Table 2).

## 3. Interaction Between Different Immune Cells in Tme

**Figure 3:**
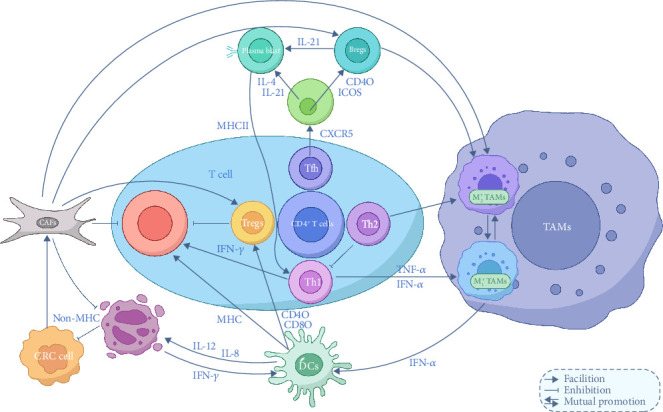
Interaction between different immune cells in TME. TME, tumor immune microenvironment.

In the TME, interactions between immune cells are crucial for both immune surveillance and immune evasion by tumors. These immune cells regulate one another through a complex network, significantly influencing tumor progression and the response to therapies (Figure 3).

### 3.1. Lymphocyte Interactions

Lymphocyte interactions in CRC create a complex immune network involving both synergy and suppression between different lymphocyte subpopulations. This network not only determines the strength and direction of the immune response but also directly influences tumor growth and metastasis. The interactions among T-cell subsets are essential in regulating antitumor immune responses. CD4^+^ T cells, including subpopulations such as Th1, Th2, and Th17, play a central supportive role through cytokine secretion and direct contact with other cells. CD8^+^ T cells, key effectors in tumor killing, are regulated by other immune cells to maintain their activity. The activation of Th1 cells is generally associated with a favorable tumor prognosis. Th1 cytokines like IFN-*γ* and TNF-*α* enhance the cytotoxicity of CD8^+^ T cells, activate M1-type TAMs, and promote their ability to phagocytose and kill tumor cells. Additionally, Th1 cytokines increase the expression of MHC class I molecules, making tumor cells more easily recognized by CD8^+^ T cells. In contrast, the cytokines secreted by Th2 cells, including IL-4, IL-5, and IL-13, can inhibit Th1 cell functions while promoting B cell activation and the production of IgE and IgG4 antibodies. This imbalance in cytokine profiles negatively affects the antitumor activity of CD8^+^ T cells, contributing to CRC progression. Other subpopulations of T cells, such as Th17 and Th22, exhibit either protumor or inhibitory effects depending on the specific conditions of the TME. In general, the inflammatory factors they produce can trigger a strong early inflammatory response, which can have an antitumor effect at the onset of the disease. However, chronic inflammation over time activates Tregs, which induce tumor immune tolerance and promote tumor progression. In addition, follicular helper T cells (Tfh), a special subset of CD4^+^ T cells, play a crucial role in the humoral immune response. Tfh cells interact with B cells by expressing the CXCR5 chemokine receptor, which allows them to localize within lymphoid follicles of secondary lymphoid organs. Tfh cells assist B cells by secreting cytokines such as IL-21 and IL-4, which promote B cell proliferation, somatic hypermutation, antibody class switching, and affinity maturation. IL-4, in particular, facilitates the conversion of IgM to IgG1 and IgE. Tfh cells also express costimulatory molecules like ICOS and PD-1, which interact with corresponding ligands on B cells to enhance B cell activation and antibody production. Notably, CD40L expressed by Tfh cells interacts with CD40 on B cells, stimulating B cell proliferation and promoting antibody class switching [198]. Furthermore, B cells, in addition to producing antibodies against tumor-specific antigens, can neutralize tumor growth factors or directly kill tumor cells through ADCC. These antibodies also function as antigen-presenting cells, presenting tumor antigens to CD4^+^ T cells through MHC class II molecules, indirectly activating CD8^+^ T cells and enhancing the overall immune response against the tumor.

This suggests that T cells and B cells do not operate independently within the TME. Instead, their interaction plays a significant role in influencing tumor development and the response to therapy.

### 3.2. Interactions Between Other Immune Cells

The primary objective of a suppressive TME is to hinder the function of effective antitumor immune cells while fostering the accumulation of immunosuppressive cells, enabling tumor cell immune escape or tolerance [199]. This process relies heavily on the intricate interactions between various immune cell types within the TME, which dynamically regulate the intensity and nature of the immune response. These interactions are mediated through the secretion of cytokines and chemokines, as well as the mutual regulation of surface molecules. Ultimately, these mechanisms determine crucial processes such as immune escape, tumor growth, and metastasis within the TME.

In the TME, both antitumor and protumor cells are recruited to varying degrees, and their proportions are influenced by the dynamic interactions between different cell populations. On the one hand, the activation of T cells remains a critical component of tumor immune surveillance and immune-killing responses, with CTLs being the predominant antitumor immune cells. The aggregation of Tregs, on the other hand, serves as the “cornerstone” for suppressing immune responses. This suggests that the direction of T-cell polarization plays a key role in determining the immune system's response pattern, ultimately influencing tumor progression or regression. DCs act as “signal transmitters” in the TME, initiating adaptive immune responses by capturing and processing “danger signals” or “self/harmless” antigens, typically within the lymph nodes. Upon encountering these signals, DCs present them as MHC complex molecules to CTLs, activating cytotoxic responses against tumor cells. Alternatively, DCs can present antigens to T helper (Th) cells, which play a role in regulating immune responses. In this way, DCs either induce an immune response (cell-mediated or antibody-mediated) or maintain immune tolerance by influencing T-cell polarization, ensuring that the immune system does not overreact. Additionally, DCs secrete cytokines that can further direct T-cell activation or suppression, thus modulating the overall immune response in the TME [153].

In addition, DCs serve as a critical link between innate and adaptive immunity by secreting cytokines such as IL-12 and IL-8. These cytokines activate NKCs, promoting the secretion of IFN-*γ*, which enhances the early tumor-killing and antiviral effects of NKCs. This, in turn, provides feedback that further promotes the maturation of DCs, improving their ability to present antigens to T cells, enhancing immune responses, and reducing immunosuppression within the TME. Moreover, NKCs can recognize tumor cells that have downregulated MHC-I protein expression, and when combined with CTLs, they help to eliminate these tumor cells, thereby playing a key role in immune surveillance and tumor clearance [143].

On the other hand, TAMs, as a crucial cluster of immune cells in the TME, play a dual role in both promoting tumor growth, metastasis, and immune escape, as well as participating in antitumor immune responses through specific mechanisms. TAMs act as a central figure in regulating the immune environment at various stages of tumor development, primarily through the secretion of cytokines and chemokines, as well as direct interactions with other immune cells [60, 68, 69, 158]. In the early stages of the tumor, TAMs are predominantly of the M1 phenotype. These M1 macrophages secrete a range of proinflammatory factors that help activate CD4^+^Th1-type T cells, thus enhancing cell-mediated immune responses. They also inhibit the aggregation of Tregs, reducing immune tolerance within the TME. Additionally, M1 TAMs promote the maturation of DCs, which are essential for antigen presentation and the activation of CTLs and NKCs. This action generates an immune-positive feedback loop, with TAMs at the center of orchestrating and enhancing the antitumor immune response [200].

While CAFs proliferate under the influence of various MMPs and profibrotic factors, they contribute to the establishment of an immune-tolerant environment, thereby further weakening the effectiveness of the immune response against tumors [201–203]. Overall, these findings underscore the intricate signaling networks among diverse cell populations in the TME, which collectively shape tumor growth, metastasis, and treatment responses. Understanding these mechanisms is vital for unraveling the complexity of the TME and advancing therapeutic strategies.

Understanding these complex interactions is crucial, as it provides key targets for the development of new immune therapeutic strategies. Breaking down the immune-suppressive environment and restoring or enhancing antitumor immune responses have consistently been central goals in cancer immunotherapy research.

### 3.3. Interplay Between Intestinal Microbiota and the CRC Microenvironment

**Figure 4:**
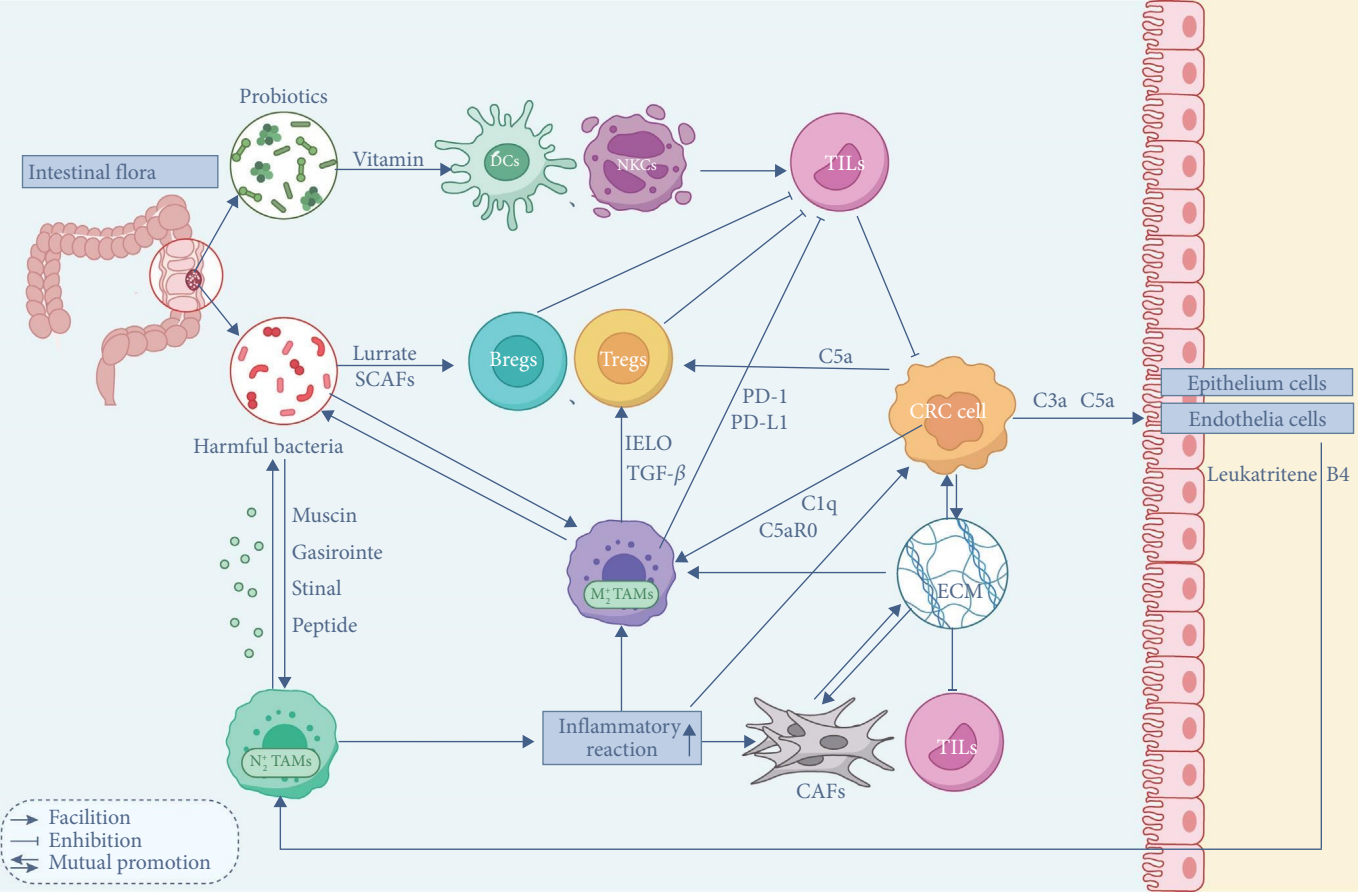
Interactions between cellular and noncellular elements in TME. TME, tumor immune microenvironment.

The diversity and balanced composition of the intestinal microbiota are essential for maintaining intestinal health. These microorganisms can be broadly categorized into three functional groups: probiotics, conditionally pathogenic bacteria, and pathogenic bacteria. Probiotics, such as Lactobacillus and Bifidobacterium lactis, contribute to host health by synthesizing vitamins, aiding digestion, and suppressing harmful bacteria. Conversely, conditionally pathogenic and pathogenic bacteria, including certain strains of *Escherichia coli* and *Fusobacterium nucleatum*, can disrupt intestinal equilibrium when overpopulated [204–206]. Such disruptions may lead to infections, carcinogen production, and an altered intestinal environment, increasing CRC risk through mechanisms like metabolic imbalance, immune dysregulation, and ecological competition [207] (Figure 4).

Currently, three hypotheses are proposed to explain the origin of the intratumoral microbiota in CRC: (a) Invasion from mucosa. (b) Migration from normal adjacent tissues (NATs). This hypothesis suggests that microbial communities in CRC tumor tissues closely resemble those in NATs, and the immunosuppressive and hypoxic TME may be more conducive to microbial colonization than NATs. (c) Invasion from the circulatory system: Oral microbiota, for instance, can enter the TME via the bloodstream and subsequently colonize CRC tumor tissues [208].

Many studies have identified unique microbial signatures in CRC patients compared to healthy matched controls, with variations influenced by factors such as patient age, disease stage, and tumor location. However, it is important to note that the correlation of specific microbial species with CRC does not necessarily indicate a direct role in tumor development or progression. It remains unclear whether changes in the microbiome drive CRC development or if these microbial alterations occur as a consequence of tumor initiation [209].

The necrotic areas of the tumor release specific chemical signals that promote the accumulation of intratumor microbiota. This colonization disrupts the immune system, helping the tumor evade various forms of immune surveillance. As a result, cancerous cells are removed from the body's immune recognition, eventually leading to abnormal and malignant proliferation [210].

The intratumor microbiota not only remodels the immune microenvironment of CRC, promoting the formation of an immunosuppressive environment and immune evasion within the TME, but it can also contribute to the development of resistance to chemotherapeutic drugs. The intratumor microbiota can thrive and proliferate within the TME, driven by prolonged nutrient-rich and highly hypoxic conditions. Notably, studies have highlighted differences in the composition of the intratumor microbiota across tissues from various tumor types [211].

In CRC patients, the gut microbiome typically exhibits pronounced ecological dysbiosis [212]. Compared to healthy individuals, CRC-associated gut flora shows greater species richness but a distinct composition, characterized by lower levels of potentially protective species like Roseburia and elevated levels of procarcinogenic species such as Bacteroides, Escherichia, *F. nucleatum*, and Porphyromonas [213]. Understanding these microbiome dynamics is critical for advancing anticancer strategies and optimizing therapeutic interventions. The gut microbiome plays a pivotal role in tumor-associated immune regulation, influencing both intestinal and systemic immune systems through multiple pathways [214]. These include the translocation of microbial components, recycling of metabolites, and immune cell migration. Variations in the colonization sites and subpopulations of gut microbes significantly impact peripheral adaptive B-cell populations, such as marginal zone B cells and plasmacytoid DCs. The presence of bacteria such as *F. nucleatum*, Bacteroides, and Treponema—which are found at higher levels in CRC—has been associated with an increased risk of CRC [215, 216]. In contrast, the enrichment of certain bacteria, like Paraburkholderia fungorum, can protect the host from cancer by influencing alanine, aspartate, and glutamate metabolism [217, 218]. In general, invasive bacteria with a “tumor-promoting” effect are predominant in the TME. These “tumor-promoting” bacteria are not randomly distributed; they are homogeneously dispersed and tend to thrive in areas with low vascularization and high immunosuppression, which align with their mechanisms within the TME. On the one hand, the intratumor microbiota contributes to the immunosuppressive environment in the TME by suppressing immune cells and immune-related factors [219]. On the other hand, the creation of an immunosuppressive and hypoxic environment provides favorable conditions for the aggregation of opportunistic pathogenic bacteria within the TME [220].

The intratumor microbiota can produce proteins that suppress the immune response, reduce the accumulation of T cells and neutrophils, and inhibit the cytotoxic activity of NKCs against both bacterial and cancer cells [219, 221]. Compared to bacteria-negative tumors, bacteria-positive tumors exhibit lower levels of CD4 and CD8 expression alongside higher expression of immunosuppressive molecules such as CTLA-4 and arginase-1 (ARG1). In contrast, the surrounding tissues of bacteria-negative tumors show higher levels of T cells and neutrophils, suggesting that the intratumor microbiota, or its metabolites, can exert a repulsive effect on immune-killing lymphocytes (T cells, neutrophils, and NK cells).

Immunosuppressive myeloid-derived suppressor cells (MDSCs) facilitate the colonization of the intratumor microbiota, especially when immune neutrophils and T cells inhibit bacterial invasion. *F. nucleatum* is one of the most prevalent components of the intratumor microbiota in the CRC TME. A significant increase in the number of monocytic MDSCs (M-MDSCs) and granulocytic MDSCs (G-MDSCs) has been observed in the TME of CRC patients enriched with *F. nucleatum*. In mice, the number of MDSCs can increase by 6.7-fold and 8.9-fold, respectively, in response to *F. nucleatum* colonization [222].

The gut microbiome can also influence cytokine production. It has been shown to alter proinflammatory IL-17 production, which may accelerate CRC progression by interacting with cells in the TME. This interaction promotes tumor cell proliferation via the extracellular signal-regulated kinase (ERK), p38 MAPK, and NF-*κ*B signaling pathways while also inhibiting CD8^+^ T cells and Tregs [223–225].

It has been reported significant differences in the composition and function of the intestinal microbiota between males and females, which could influence immune function and metabolic processes in the gut mucosa, thereby affecting CRC risk [226]. In addition, the intestinal microbiota can also impact the host's sensitivity to, and resistance against, chemotherapeutic agents, potentially contributing to sex-specific variations in CRC treatment outcomes and prognosis [227].

Additionally, the gut microbiota remotely regulates lymphocyte development and homeostasis, affecting lymphocyte migration and function. It can also suppress TIL activation by modulating the maturation and functionality of DCs and NKCs [228, 229].

A critical aspect of this regulation involves metabolites produced by gut microbes. Short-chain fatty acids (SCFAs), such as butyrate generated from the anaerobic fermentation of dietary fibers, enhance the production of mucins and gastrointestinal peptides, fortify the intestinal barrier, modulate inflammatory responses, activate Tregs and B cells, and promote immune tolerance [230]. These metabolites also act as active regulators of TANs, influencing their inflammatory activity and aging phenotype throughout their lifecycle, from activation to maturation [231–233]. However, dysregulated activation of N2-type TANs exacerbates local inflammation, indirectly stimulating the activation and proliferation of CAFs, thereby accelerating tumor progression [232]. Immune cells and intestinal commensal flora share a bidirectional relationship, with each influencing the other's composition and function. Notably, TANs can alter the gut microbiota, particularly during inflammatory states, fostering the proliferation of pathogenic bacteria and the decline of probiotics. Additionally, macrophage activation is a critical factor in gut flora-associated CRC [234, 235]. For instance, lipopolysaccharides from gut microbiota can trigger the accumulation of monocyte-like macrophages, driving chronic tumorigenic inflammation and promoting colitis-associated tumorigenesis. Research has shown that depleting macrophages can eliminate the tumor-promoting effects of dysregulated gut microbiota.

During CRC progression, M2-type TAMs selectively recruit protumorigenic bacteria, such as *Clostridium perfringens*. In contrast, other bacterial species like *Helicobacter pylori* can counter tumor progression by reducing M2-type TAM infiltration and downregulating proinflammatory and tumorigenic cytokines, including TNF-*α*, IL-1, and IL-23. Efforts to harness the gut microbiome in CRC prevention and treatment are ongoing. Studies suggest that long-term probiotic supplementation increases the abundance of beneficial bacteria like Lactobacillus, restores intestinal structure, and enhances immune function. Advances in genetic engineering have enabled the modification of gut bacteria to increase microbial diversity, improving the responsiveness of ICB-resistant patients and reducing immunotoxicity. These interventions have shown the potential to boost treatment efficacy and improve patient outcomes, highlighting the gut microbiome as a promising target for novel CRC therapies and immunomodulation strategies [236–238].

## 4. Conclusion

TME of CRC is a complex and dynamic ecosystem that supports tumor survival, proliferation, infiltration, and metastasis through the intricate interactions of various immune cell types. This environment plays a critical role in both tumor progression and treatment responses. As research into CRC immunology advances, it becomes evident that the type, quantity, distribution, and functional status of both antitumor and tumor-promoting immune cells are continually evolving, with each subpopulation exerting a significant influence on tumor development. Innovations in immune cell therapies and combination treatments are increasingly focused on harnessing the unique characteristics of the TME. Therefore, studying the TME in CRC not only provides valuable insights into tumor biology but also forms the foundation for developing personalized and precise immunotherapy strategies. With continuous scientific advancements and deeper understanding, we expect significant breakthroughs in CRC immunotherapy, leading to improved treatment outcomes and enhanced quality of life for patients.

## Figures and Tables

**Table 1:** Constituents and functions of the TME cell components in CRC.

Cell type	Cell subtype		Surface marker	Secretory factor	Function
TILs					
T cells [14, 19, 20, 27, 39]	CD8^+^T cell	CD8^+^Tem	CD8, CD45RO, CD62L-	Granzyme B, perforin, IFN-*γ*, IL-2, IL-10, IL-4, TNF-*α*	Exerting antitumor effects
		CD8^+^Trm	CD8, CD103, CD69, CD49a	Granzyme B, perforin, IFN-*γ*, IL-2, IL-17, TNF-*α*, GM-CSF	Exerting local antitumor effects
		CD8^+^Trm-mitiotic	MKI67, TOP2A, STMN1	Proinflammatory factor, PD-1	Synergistic tumor promotion, portends a dismal prognosis
		CD8^+^IEL	T-cell receptor, CD8, CD69, CD45RO, CD103, CD160	Granzyme, perforin, IFN-*γ*, IL-10, IL-17, TGF-*β*, EGF, NGF	Inhibition of tumor development and maintenance of gastrointestinal homeostasis
	CD4^+^T cell	CD4^+^Tregs	CD4, Foxp3, CD25 (IL2RA), CTLA-4	IL-10, TGF-*β*, PD-1	Fulfillment of a protumorigenic role
		CD4^+^Th	CD4, CD40LG, IL7R	IL-2, IL-4, IL-17, IFN-*γ*	Exerting antitumor effects
		CD4^+^Th-1-like	CD4, CD40LG, CXCL13	IFN-*γ*, TGF-*β*	Exerting antitumor effects
		CD4^+^Prolif	CD4, CD69(early stage), CD25(IL2RA)	IL-2, IL-4, IL-1	Exerting antitumor effects
		CD4^+^Th2	IL-3R, IL-4R, IL-5R, CCR4, CD25 (IL2RA), CD69	IL-4, IL-5, IL-9, IL-10, IL-13	Synergistic tumor promotion
B cells [45, 46, 52–54]	Naive B cell		CD19, CD20	IL-2, IL-4, IL-5, IL-6	Identify, capture tumor antigens
	Class-switched memory B cell		CD19, CD45RO, CD27, CD20	Antibody (IgG, IgA, IgE), IL-10, IL-21, TGF-*β*, cellular adhesion molecules (CD40L, ICAM-1)	Synergistic antitumor
	Plasmablasts		CD19, CD24, CD38	IgM, IgG, IgA, IgE, IL-10, IL-6, TGF-*β*, CD27, CD38	Exerting antitumor effects
	Regulatory B cell		CD20, CD73	IL-10, IL-35, TGF-*β*, complement factor (C1q, C3, C4)	Synergistic tumor promotion

BMDCs					
TAMs [57–59]	M1 type TAMs		CD68, CD86, CD80, CD14, MHCⅡ, iNOS, ROS, RNS	IL-6, IL-12, IL-23, IL-1*β*, TNF-*α*	Exerting antitumor effects
	M2 type TAMs	M2a	CD68, CD163, CD206, CD14, CD16, CD31, MHCⅡ	IL-1ra, sIL-1R, IL-10, CCL17, CCL18, CCL22, CCL24, EGF, TGF-*β*, IGF1, Arg1	Fulfillment of a protumorigenic role
		M2b	CD68, CD86, CD14, CD16, MHCⅡ	IL-1β, IL-6, IL-10, TNF-*α*, CCL1, CCL20, CXCL1, CXCL3, COX-2	Fulfillment of a protumorigenic role
		M2c	CD68, CD150, CD163, CD206, CD14, CD16	IL-10, CXCL13, CCL16, CCL18, CCR2	Fulfillment of a protumorigenic role
		M2d	CD68, CD14, CD16	IL-10, IL-12, CXCL10, CDXL16, CCL5, VEGF, iNOS, TGF-*β*	Fulfillment of a protumorigenic role
DCs [76, 81, 83]	Immunogenic DCs	cDC1s	CLEC9A (DNGR-1), CD11c, XCR1, CD141 (BDCA-3),	IL-12, IFN-1, TNF-*α*	Synergistic both antitumor/tumor promotion
		cDC2s	CD11c, CD11b, CD1c (BDCA-1), CD172a (SIRP*α*)	IL-1*β*, IL-6, IL-23	Synergistic antitumor
	Immunosuppressive DCs	Myeloid-derived suppressor cells (MDSCs)	CD11b, CD33, Gr-1, HLA-DR	Arg-1, IDO, TGF-*β*	Fulfillment of a protumorigenic role
		Regulatory dendritic cells (DCregs)	PD-L1, PD-L2, CD80, CD86, CD11b, CD11c	IL-10, TGF-*β*, IDO	Fulfillment of a protumorigenic role
		Plasmacytoid dendritic cells (pDCs)	CD80, CD86, CD123 (IL-3Rα), CD303 (BDCA-2), CD304 (BDCA-4)	IFN-*α*, TNF-*α*, IL-6	Synergistic tumor promotion
		Tolerogenic DCs (tolDCs)	CD11c, CD80, CD86, PD-L1, PD-L2, CD83	IL-10, TGF-*β*, Indoleamine 2,3-dioxygenase (IDO)	Synergistic tumor promotion
	Plastic DCs	Langerhans Cells	CD1a, CD11c, HLA-DR, Langerin (CD207)	IL-10, IL-12, TNF-*α*	Synergistic both antitumor/tumor promotion
		Monocyte-derived DCs (MoDCs)	CD80, CD86,CD11b, CD11c, HLA-DR	IL-10, IL-12, TNF-*α*	Synergistic tumor promotion
NKC [100, 101]	Activated NKC		CD56, CD69, CD25, CD137(4-1BB), NKG2D, HLA-DR	IFN-*γ*, TNF-*α*	Exerting antitumor effects
	Immunosuppressed NKC		PD-1, CTLA-4, lymphocyte activating gene-3 (LAG-3), T-cell immunoglobulin and mucin-binding protein-3 (Tim-3)	IL-10, TGF-*β*	Exerting antitumor effects
Granulocyte	TANs [124, 125]	N1 type TANs	CD177, CD66b	IL-1*β*, IL-12, CXCL9, CXCL10, TNF-*α*, ROS	Exerting antitumor effects
		N2 type TANs	Recognition type oxidized low-density lipoprotein receptor-1 (LOX-1), CXCR4	IL-10, TGF-*β*, IL-6, IL-17, IL-8, IL-1*β*	Synergistic tumor promotion
	TIEs [126]	N1 type TIEs	CD69, CD11b	IL-12, IFN-*γ*, TNF-*α*	Synergistic antitumor
		N2 type TIEs	CD39, CD163	IL-10, TGF-*β*, IL-6	Synergistic tumor promotion
	TIBs [127]		CCR3, CD11b, Siglec-8	IL-4, IL-5, IL-13	Synergistic tumor promotion
MSCs [131–133]	–		CD90, CD44, CD73	TGF-*β*, SDF-1, IL-6	Synergistic tumor promotion

Others					
CAFs	Activated CAFs		Alpha-smooth muscle actin (*α*-SMA), fibroblast specific protein 1(FSP1)	TGF-*β*, matrix metallo-proteinases (MMP)	Synergistic tumor promotion
	Fatty plasmid-enriched CAFs		Uncertain, possibly associated with adipose plasmid-related proteins	Adiponectin, aliphatic acid	Synergistic tumor promotion
	Inflammatory CAFs		Uncertain, possibly associated with inflammation-related cell surface molecules	IL-6, IL-8, IL-1*β*	Synergistic tumor promotion
	Immunosuppressive CAFs		Uncertain, possibly associated with immune regulation -related cell surface molecules	IL-10, TGF-*β*	Synergistic tumor promotion
	Vascular CAFs		Uncertain, possibly associated with angiogenesis-related cell surface molecules	VEGF, Basic fibroblast growth factor (bFGF)	Synergistic tumor promotion
Endothelial cells	Continuous endothelial cells		CD31, CD34	VEGF, angiotensin	Synergistic antitumor
	Discontinuous endothelial cells		Endoglin, vascular endothelial cadherin	VEGF, FGF	Synergistic tumor promotion
	Tumor-associated endothelial cells (TECs)		Endoglin, CD105, VEGFR-2	VEGF, PDGF, TGF-*β*, TNF-*α*	Synergistic tumor promotion
Adipocyte	White adipocytes		CD36, adiponectin receptor	Nonesterified fatty acid, glycerinum, adiponectin, TNF-*α*, IL-6, IL-8	Synergistic tumor promotion
Nerve cell	Peripheral neurons/sympathetic and parasympathetic neurons/neuroendocrine cells		Neurofilament, neurotrophic receptors (TrkB, TrkC, p75NTR)	Neurotransmitter (acetylcholine, norepinephrine), neurotrophic factor (NGF, BDNF), neuroendocrine substance (5-hydroxytryptamine, adrenaline)	Synergistic both antitumor/tumor promotion

**Table 2:** The distinct immune features of MSS and MSI CRC.

Features [142, 143]	MSS CRC	MSI CRC
Mutation burden	Low	High
Immunogenicity	Weak	Strong
Immune cell Infiltration	Limited	Abundant
Immune suppressive cells	High	Low
Immune-related gene expression	Low	High
Immune checkpoint Inhibitor efficacy	Poor	Good
Tumor microenvironment type	Immune-cold	Immune-hot

## Data Availability

The data used in this review are available from the authors upon reasonable request.
